# Dynamic, but Not Necessarily Disordered, Human-Virus Interactions Mediated through SLiMs in Viral Proteins

**DOI:** 10.3390/v13122369

**Published:** 2021-11-26

**Authors:** Heidy Elkhaligy, Christian A. Balbin, Jessica L. Gonzalez, Teresa Liberatore, Jessica Siltberg-Liberles

**Affiliations:** 1 Department of Biological Sciences, Florida International University, Miami, FL 33199, USA; helkh002@fiu.edu (H.E.); cbalbin@fiu.edu (C.A.B.); jgonz750@fiu.edu (J.L.G.); tlibe003@fiu.edu (T.L.); 2 Biomolecular Sciences Institute, Florida International University, Miami, FL 33199, USA

**Keywords:** short eukaryotic linear motifs, SLiMs, viral-host protein interaction, intrinsically disordered protein regions, the ELM database

## Abstract

Most viruses have small genomes that encode proteins needed to perform essential enzymatic functions. Across virus families, primary enzyme functions are under functional constraint; however, secondary functions mediated by exposed protein surfaces that promote interactions with the host proteins may be less constrained. Viruses often form transient interactions with host proteins through conformationally flexible interfaces. Exposed flexible amino acid residues are known to evolve rapidly suggesting that secondary functions may generate diverse interaction potentials between viruses within the same viral family. One mechanism of interaction is viral mimicry through short linear motifs (SLiMs) that act as functional signatures in host proteins. Viral SLiMs display specific patterns of adjacent amino acids that resemble their host SLiMs and may occur by chance numerous times in viral proteins due to mutational and selective processes. Through mimicry of SLiMs in the host cell proteome, viruses can interfere with the protein interaction network of the host and utilize the host-cell machinery to their benefit. The overlap between rapidly evolving protein regions and the location of functionally critical SLiMs suggest that these motifs and their functional potential may be rapidly rewired causing variation in pathogenicity, infectivity, and virulence of related viruses. The following review provides an overview of known viral SLiMs with select examples of their role in the life cycle of a virus, and a discussion of the structural properties of experimentally validated SLiMs highlighting that a large portion of known viral SLiMs are devoid of predicted intrinsic disorder based on the viral SLiMs from the ELM database.

## 1. Introduction

Viruses are pathogens that cannot thrive outside a host [1,2]. Depending on the viral family, genomic information is encoded in either positive or negative single-stranded or double-stranded DNA or RNA. The genomic material is typically small, ranging from a few kb to over 1000 kb [3]. Viruses exploit host cell proteins to complete their life cycle: attachment, penetration, uncoating, replication and protein expression, assembly, and egress from the infected cell [1]. The viral genome is translated into structural proteins, non-structural proteins, and sometimes accessory proteins. Structural proteins encapsulate the newly formed virus genome inside the host cell and provide the virion its shape. Non-structural proteins (nsps) typically make up the genome replication complex and include a polymerase that is dedicated to replicating the viral genome. Further, nsps partake in protein processing and may also perform secondary functions involved in impacting immune regulation and antiviral response. Accessory proteins are mainly regulatory proteins primarily involved in modulating host cell gene expression, inducing apoptosis, or affecting the viral rate of replication [4]. 

Viruses have high mutation rates [5], which is particularly true with regard to RNA viruses [6]. The fitness of RNA viruses depends on their RNA polymerases to replicate the viral genome with low fidelity [7,8]. While the primary enzymatic functions typically are under selective constraint, rapidly evolving amino acid residues are often located in conformationally flexible regions on the surface of the protein. Surfaces of viral proteins are major contact points to their hosts. Through interface mimicry, where a part of a viral protein surface resembles a host protein, the virus can interfere with protein-protein networks of the host protein [9]. The presence of short linear motifs (SLiMs) that act as functional signatures in proteins are important for understanding protein-protein interactions in an organism. Identification of a SLiM from a host species in a viral protein suggests interface mimicry that may disrupt endogenous protein-protein interactions. Many host-virus mimicry-driven interactions are transient [10] and depend on the proteomic context of the host cell. Consequently, exogeneous interactions may give rise to complex diversity in virulence, pathogenicity, and transmissibility not only between different host species, but also within the same host species. 

### 1.1. Short Linear Motifs 

Eukaryotic Linear Motifs (ELMs) (a.k.a. SLiMs) are small segments of proteins, usually 3 to 10 amino acids long with a specific cellular function [11,12]. Given the linear sequence pattern that composes a SLiM, some positions in a SLiM can withstand various amino acid substitutions without affecting functionality, while an amino acid substitution at a different, critical position can eliminate all functionality. To represent sequence variation, SLiMs are described by regular expressions using the one-letter amino acid abbreviations [13]. Virus proteins that display SLiMs can perform molecular interactions with host proteins in a similar manner as the host protein it mimics [11]. SLiMs that occur in humans may also occur by chance in viral proteins due to convergent evolution [10]. SLiMs can occur in highly conserved protein regions or regions with a high evolutionary rate of amino acid substitution. The presence of conserved motifs within the same virus family suggests the existence of functionally important virus-host protein interactions. Conversely, the presence of rapidly evolving motifs can enable the emergence of new protein-protein interactions within different hosts [11,14]. 

### 1.2. SLiMs in Intrinsically Disordered Protein Regions 

Intrinsically disordered regions (IDRs) lack a specific folded structure (order) and harbor high conformational plasticity [15]. Linear motifs from eukaryotes were found to be predominantly disordered based on prediction of intrinsic disorder [16]. Viral motifs within intrinsically disordered protein regions (IDRs) can enable viral-host protein interactions [2,11,12]. IDRs provide SLiMs malleability to interact with various target proteins and to acquire different transient secondary structures that facilitate SLiM interaction with another protein [11,15,17,18,19]. The plasticity of SLiMs has been proposed to impact viral phenotypic traits such as tropism and virulence [20]. 

A positive correlation between disorder content and the occurrence of linear motifs has been shown [11]. However, disorder content has been found to vary greatly between virus families and coronaviruses have among the least [21]. Proteome-wide evolutionary studies of coronaviruses revealed a highly disordered nucleocapsid protein while the other proteins had almost no disorder [22]. Yet, from the large SARS-CoV-2 data that has been accumulating over the last two years, it is apparent that coronaviruses like SARS-CoV-2 perform a wealth of interactions with proteins in its human host despite a low predicted intrinsic disorder content.

## 2. Methods Used in the Discovery of SLiMs

### 2.1. Experimental Procedures

SLiMs are typically involved in transient protein-protein interactions (PPIs) with a low affinity towards the interacting protein [23,24]. Thus, mass spectroscopic analysis of PPIs might be unable to detect the SLiMs’ temporary interactions in their normal mode; more specific optimizations are needed [25]. Other methods that have been proposed for the discovery and investigation of SLiM interactions are peptide phage display and large-scale proteomic peptide phage display [26]. Phage display may be coupled with site-directed mutagenesis to verify the interacting pattern. One major disadvantage of the experimental methods exploring SLiMs on the peptide level is that the actual interaction inside the cell might not be properly portrayed due to the absence of post-translational protein modifications that are critical for the functionality of the SLiM [26]. 

### 2.2. Computational Approaches 

Data from experimentally verified SLiMs can be used to make predictors or search functions for similar motifs. Various webservers with databases of linear motifs provide a search function for similar motifs using regular expression patterns (regex). According to the ELM database [27], the regex pattern symbols used are as follows: dot “.” means that this position permits the presence of any amino acid which can be symbolized by “x” as well, square brackets “[ ]” mean any listed amino acid is accepted at that position, caret sign inside a square bracket ”[^]” means that any following amino acid is not allowed in this site, curly brackets “{ }” specify the count or range of accepted amino acids at specific position in the pattern, dollar sign “$” indicates the C-terminal end of the protein sequence, caret sign “^” indicates the N-terminal end of the protein, question mark “?” indicates one optional amino acid (one or none), asterisk “*” specifies any number of optional amino acids is allowed (zero or more), plus sign “+” indicates one or more amino acids are accepted, pipe “|” separates and suggests an alternative amino acid pattern for the motif, and parentheses “( )” can either be used to group pieces of pattern or to indicate an important amino acid site such as covalently modified amino acids. 

The ELM database is the prevalent resource for SLiMs. This database provides experimentally verified SLiMs classified as true positives [27]. SLiMs are categorized by function as either cleavage, degradation, docking, ligand binding, modification, or targeting sites [27]. Cleavage sites (CLV) are patterns identified by different proteolytic enzymes. Degradation sites (DEG) are sequences recognized for ubiquitination to allow subsequent protein breakdown. Docking sites (DOC) are involved in regulating protein interaction. Ligand binding sites (LIG) participate in protein-protein interactions. Modification sites (MOD) include amino acid patterns predicted to undergo post-translational modification. Targeting sites (TRG) act as signals for translocation of proteins [12,27]. 

Other resources are available, for instance SLiMSearch and MEME suite. SLiMSearch is a webserver that allows the user to input a regex pattern or motif consensus sequence and then choose the species where the motif is predicted to be found, along with other filtration options such as disorder cutoff value. The results provide proteins that potentially include the input motif with their predicted conservation score, relative disorder score, accessibility prediction, PTM predictions at the motif site, the presence of known, mutational SNPs in that region, and more data that can allow the user to filter the results based on their needs [28]. MEME suite includes many tools and pipelines for *de novo* motif discovery and searching for known motif patterns in your input dataset as well as performing enrichment analyses and more [29]. 

A critical challenge for the computational techniques is their high false-positive rate [12,30,31]. Filtration to reduce false positives include ensuring the SLiM is in a disordered region is commonly recommended and integrated in some tools like SLiMSuite [32] and IUPRED3 [33].

## 3. Are Viral SLiMs Disordered?

SLiMs from the ELM database were shown to be disordered using *m*ean *I*UPRED *d*isorder *s*cores (MIDS) [16,34]. IUPRED predicts a disorder score for amino acid residues in proteins [35,36]. If the score for a residue is greater than 0.5, that residue is predicted to be disordered. However, a cutoff of 0.4 has been shown to be in greater agreement with experimentally confirmed intrinsic disorder [16]. Considering a 0.4 cutoff, 78% [16] and 71% [34] of all motifs were found to have a MIDS above 0.4 indicating that some residues in some motifs are likely ordered.

To the best of our knowledge no study has investigated the viral SLiMs separately. With the large variation in disordered content in virus families [21], we were curious about the disorder content in viral SLiMs. To investigate the disorder content of linear motifs from viruses that are known to interact with host proteins, we undertook a brief study in that respect. We downloaded the FASTA sequences for all 260 viral SLiMs classified as true positives from the ELM database [27]. This dataset contains 131 LIG, 65 MOD, 38 TRG, 11 DOC, 11 CLV, and 4 DEG viral SLiM sites. For each sequence, we extracted the motif plus 50 flanking amino acid residues on the N-terminal and C-terminal sides, respectively. For sequences where the motif was located closer than 50 amino acid residues from a terminal, all residues towards that terminal were included. The resulting sequence fragments were used to predict intrinsic disorder with IUPRED2 [35,36] (default settings). The predicted state was mapped to the corresponding position in each sequence using an IUPRED disorder score cutoff of 0.4 (and 0.5 separately) to infer disorder or order. Thereafter, the percentage of disordered residues for each motif region was calculated. We also calculated MIDS per motif. 

**Figure 1:**
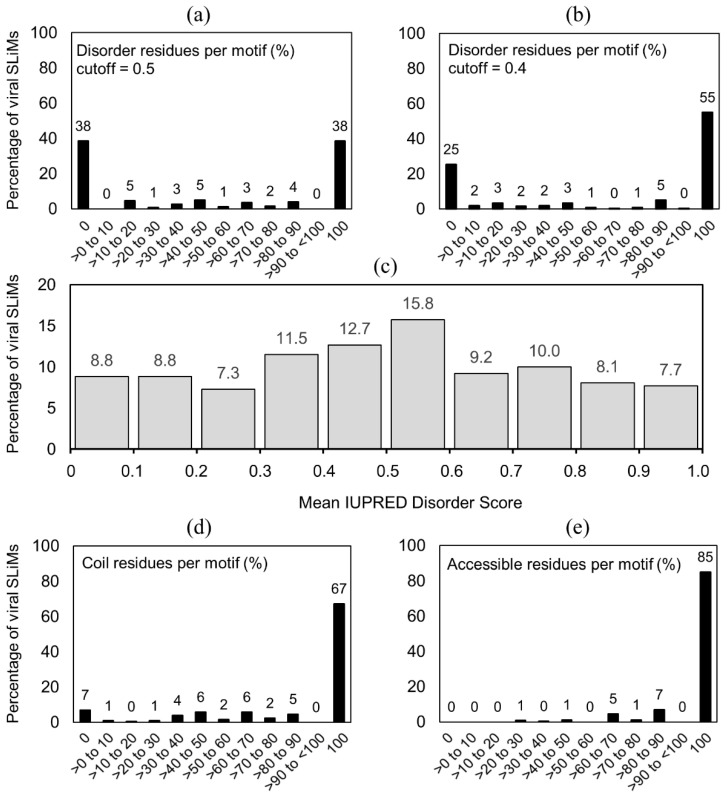
Predicted structural features of 260 viral SLiMs from the ELM database. The percentage of viral motifs with a certain disorder content as inferred from IUPRED prediction using a cutoff of (**a**) 0.5 and (**b**) 0.4. (**c**) The percentage of viral motifs with a certain Mean IUPRED Disorder Score (MIDS). The percentage of viral motifs with a certain (**d**) secondary structure (coil) and (**e**) surface accessibility content as inferred from NetSurfP-2.0 prediction. The percentages shown are approximate; rounded to the nearest whole number for a, b, d, and e, and to the nearest tenth for c. See also Table S1.

We found that 38% of the viral motifs are completely disordered and another 38% are completely ordered based on IUPRED disorder scores with cutoff = 0.5. For the remaining motifs, disorder content varies (Figure 1a). Based on IUPRED disorder scores with cutoff = 0.4, 66 motifs (25%) are 100% ordered and 143 motifs (55%) are 100% disordered (Figure 1b). The predominant motif classes vary between the fully ordered and the fully disordered motifs. Of the fully disordered motifs, the predominant motifs are LIG (63%) and TRG (18%). Of the fully ordered motifs, the predominant motif classes are MOD (62%) and LIG (15%). MIDS revealed that >36% of all viral motifs had an average score below 0.4 (Figure 1c). These results suggest that screening for only disordered motifs may exclude a large portion of functional viral motifs and especially sites that undergo post-translational modification. 

Further, we also predicted surface accessibility and secondary structure for the 260 viral motifs with NetSurfP-2.0 [37] with default settings. The NetSurfP-2.0 predictions were used to infer “not alpha helix or beta strand” as coil and surface accessibility for each residue in the motif. Thereafter, the fraction of coil and surface accessible residues for each motif region was calculated. Most motifs are as expected surface accessible and tend to lack secondary structure. From the 260 viral motifs, 175 motifs (67%) are 100% coil, and 221 motifs (85%) are completely surface accessible (Figure 1d,e).

Based on prediction of disorder, surface accessibility, and secondary structure, our results suggest that a large portion of the true positive viral SLiMs are not disordered but a clear majority are in a coil conformation and an even stronger signal is seen from prediction of surface accessibility. Ultimately, these results, based on predictions of a limited set of viral linear motifs known to interact with host proteins, imply that viral SLiMs may not be as disordered as their analogous counterparts in eukaryotes. Further analyses are warranted to establish how disorder content varies for the same SLiM in a virus and its host. Here, we show selected examples of SLiMs that illustrate how disorder, surface accessibility, and secondary structure may vary across related viruses.

## 4. Select Viral SLiMs Involved in the Viral Life Cycle

**Figure 2:**
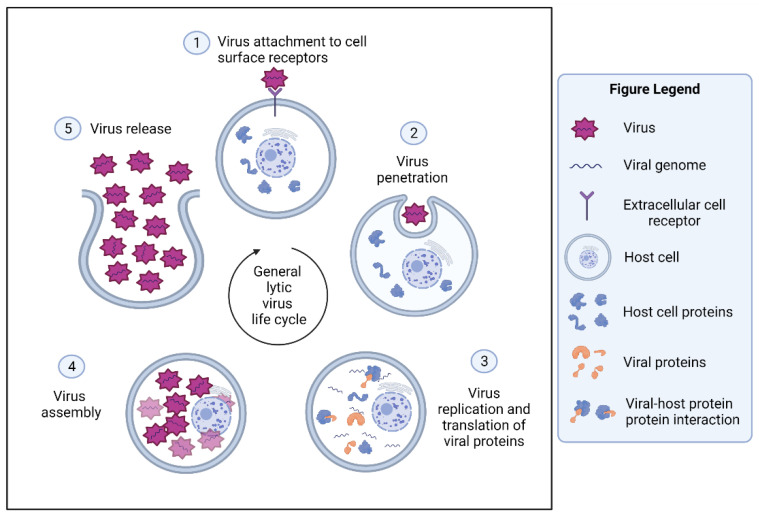
The general lytic virus life cycle inside the cells. (1) The virion attaches to the cell surface receptors. (2) The penetration of the virus through endocytosis to the infected cell. (3) The replicated genome and translated viral proteins inside the cell. (4) The newly assembled viruses inside the cell. (5) The cell lysis and release of new viruses from the infected cell. Created with BioRender.com (accessed on 30 October 2021).

The viral life cycle can be divided into events that occur outside the cell and inside the infected cell. In a general viral lytic cycle (Figure 2), the virus must first attach and fuse to the outside of the host cell before it can enter the cell. Then, the virus gets encapsulated or penetrates the cell membrane. Next, the virus starts the process of replication and translating its proteins to produce more viruses that are capable of infecting other neighboring cells. At this step, viral proteins hover inside the cell and migrate to several subcellular locations. As for host proteins, the presence of SLiMs in viruses may aid in the shuttling of viral proteins to different cellular compartments, where they can interact with various host proteins [27]. Finally, the virus particles are assembled, followed by viral exit from the infected cell [1]. 

### 4.1. SLiMs and Viral Cell Invasion through Cellular Attachment, Entry, and Fusion

#### 4.1.1. RGD Motif, Integrin-Binding, and Attachment

The existence of specific motifs can enhance the ability of a virus to attach to the host cell receptors. For instance, the presence of the RGD pattern in virus envelope or membrane proteins, such as for Foot and Mouth disease virus (FMDV) [38] and Epstein-Barr virus [39], may promote viral fusion with host cells by facilitating the interaction with the integrin cell surface receptors [40]. Integrin receptors are transmembrane receptors that are involved in various signaling pathways including cellular communication with the surrounding environment. Several cell types, such as pneumocytes, endothelial cells, and platelets, express integrin transmembrane receptors. When transmembrane integrin receptors recognize and bind to a pattern of RGD amino acids present on extracellular proteins, it can result in activation or inhibition of the integrin receptor’s signaling pathways [41]. RGD integrin-binding activates clathrin-mediated endocytosis in adenoviruses and promotes virus entry into cells, triggering the phosphatidylinositol-3-kinase (PI3K) and mitogen-activated protein kinase (MAPK) pathways inside the infected cells. PI3K and MAPK are critical signaling pathways that control cell survival and proliferation [42].

The spike receptor-binding domain (RBD) from SARS-CoV-2 has an RGD motif that thus far is not found in other closely related coronaviruses [43]. The motif shows a degree of structural resemblance to other experimentally confirmed RGD-containing ligands and proteins that can bind to integrin receptors. Although the motif is not completely solvent accessible, it is located near a disordered protein region which may expose the RGD motif in a subset of the conformational ensemble enough to enable integrin binding under some conditions [44]. It has been speculated that the RGD motif could (1) promote the entry of SARS-CoV-2 into cells not expressing the primary SARS-CoV-2 receptor, the ACE2 receptor [45], and (2) affect the infectivity of the SARS-CoV-2 virus [43,44] due to the conformational flexibility surrounding the motif [44].

#### 4.1.2. Furin Cleavage Motif Role in Viral Entry 

To enhance cell entry, numerous viruses use a motif of the furin recognition pattern. Furin is a ubiquitously expressed protease [46] that promotes splitting and activation of various human extracellular proteins including hormones, growth factors, cellular receptors, adhesion molecules, and more [47]. Furin recognition patterns, R.[RK]R., where furin cleaves the protein after the last Arginine (R) in the pattern, have been confirmed experimentally in HIV-1 [48], Coronaviruses [49], Flaviviruses [50], and other viruses (discussed in [47]), and in some bacterial toxins such as Anthrax toxin [51] and Diphtheria toxin [52]. 

In viruses, furin cleavage can lead to activation and facilitation of the viral fusion to cellular receptors and cell entry [53,54]. In Flaviviruses, furin proteolysis of precursor membrane (prM) protein is required to develop mature viruses [55]. In Orthomyxoviruses, such as influenza viruses, hemagglutinin (HA) glycoprotein cleavage leads to activation of the virus by unveiling the fusion peptide responsible for cell fusion and entry [56]. HA cleavage in avian influenza viruses was found responsible for the increased pathogenicity [53]. 

**Figure 3:**
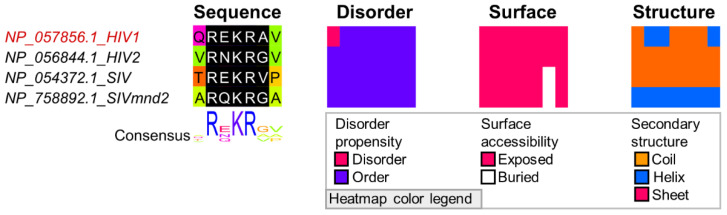
The furin cleavage site in the envelope glycoprotein from HIV. Sequences were identified with BLAST using the envelope protein (accession: NP_057856.1) from HIV-1 as query. Sequence names shown in red represents true positive instances from the ELM database [27]. The multiple sequence alignment (MSA) was built with MAFFT+L-INS-i [57] in Jalview [58]. The regular expression pattern R.[RK]R. from motif CLV_PCSK_FUR_1 in the ELM database [27] was identified using Find in Jalview, shown in black with white text. The region shown under Sequence shows the amino acids that corresponds to the true positive motif from ENV_HIV1 plus one additional site on each side. The three additional heatmaps display the same region of the alignment colored by property. The heatmap for Disorder propensity displays disordered (magenta) or ordered (purple) residues based on IUPRED prediction with cutoff = 0.4 [35,36,59]. Heatmaps for (1) Surface accessibility displays surface exposed (magenta) and buried (white) residues and (2) Secondary structure displays coil (orange) and secondary structure (helix: blue, strand: magenta) based on NetSurfP-2.0 predictions.

The conservation of sequence, disorder, and accessibility of the furin cleavage motif in HIV-1 [48] is high across sequences of HIV-1 envelope homologs suggesting a conserved function (Figure 3).

In SARS-CoV-2, an additional furin cleavage site, absent in other closely related coronaviruses, was detected in the spike protein using sequence-based methods and it was suggested to be one of the principal causes of its pathogenicity [60]. Later, it was shown that while furin plays a role in successful SARS-CoV-2 infection, it is not critical for infection [61]. Further, other coronaviruses such as SARS-CoV also include furin recognition sites in nearby regions, and some of them were experimentally verified to be functional [62], which suggests that the exact position is not always critical for an analogous function.

### 4.2. SLiMs Influencing Viral Cell Replication 

#### 4.2.1. Retinoblastoma-Binding LxCxE Motif 

After viruses invade the host cell, the viral genome is unpacked, and genome replication is initiated. For viral replication to occur, tampering with the host cell machinery is often achieved by promoting degradation of host proteolytic enzymes responsible for breaking down virus proteins, inhibiting degradation of host proteins essential for virus survival, and altering the host cell cycle by forcing the cell to the S phase [2]. Viruses may induce host cells to the S phase to facilitate their replication through the RB-binding LxCxE motif. Retinoblastoma proteins (RBs) are tumor suppressor proteins that inhibit the G1 to S cell cycle phase transition, hindering DNA replication and cell division. DNA viruses, such as adenoviruses, human papillomaviruses, and human cytomegalovirus (HCMV), produce proteins containing the LxCxE motif that can either degrade the RB protein or inhibit its function, which will help the virus benefit from the host’s replication enzymes to replicate its genome [63,64,65].

#### 4.2.2. G3BP Protein Binding Motif

The Ras GTPase activating protein SH3 domain-binding proteins, known as G3BP, are important for viral replication. The G3BP proteins form a complex and bind to RNA when cells are under environmental stress or viral attack. Upon binding of G3BP to RNA, stress granules are formed to help the cell eliminate the virus and control the viral infection [66]. According to the ELM database, the G3BP binding motif has the pattern [FYLIMV].FG[DES]F [27], often simplified to FGDF. Human herpesvirus [67], Sindbis virus [68,69], Semliki Forest virus [70], and Chikungunya virus [70,71] include FGDF motifs capable of interacting with G3BP and altering its function. The G3BP functional alteration is essential for intracellular viral replication and overcoming the cellular antiviral response [66,67,69,70]. 

Chikungunya virus, an arbovirus that needs a mosquito vector to be transmitted to a vertebrate host, has two important FGDF motifs in the hypervariable region located towards the C-terminus of nsp3 protein. It has been shown that one FGDF motif is enough to infect the mosquito, but two FGDF motifs are necessary for the virus to be transmitted from mosquito saliva to the vertebrate host [72]. In a relative of Chikungunya virus, Semliki Forest virus, the C-terminal FGDF motif in nsp3 protein is also found to be essential for the interaction with G3BP protein, and without this motif the interaction between G3BP and the replication complex is inhibited [70]. 

**Figure 4:**
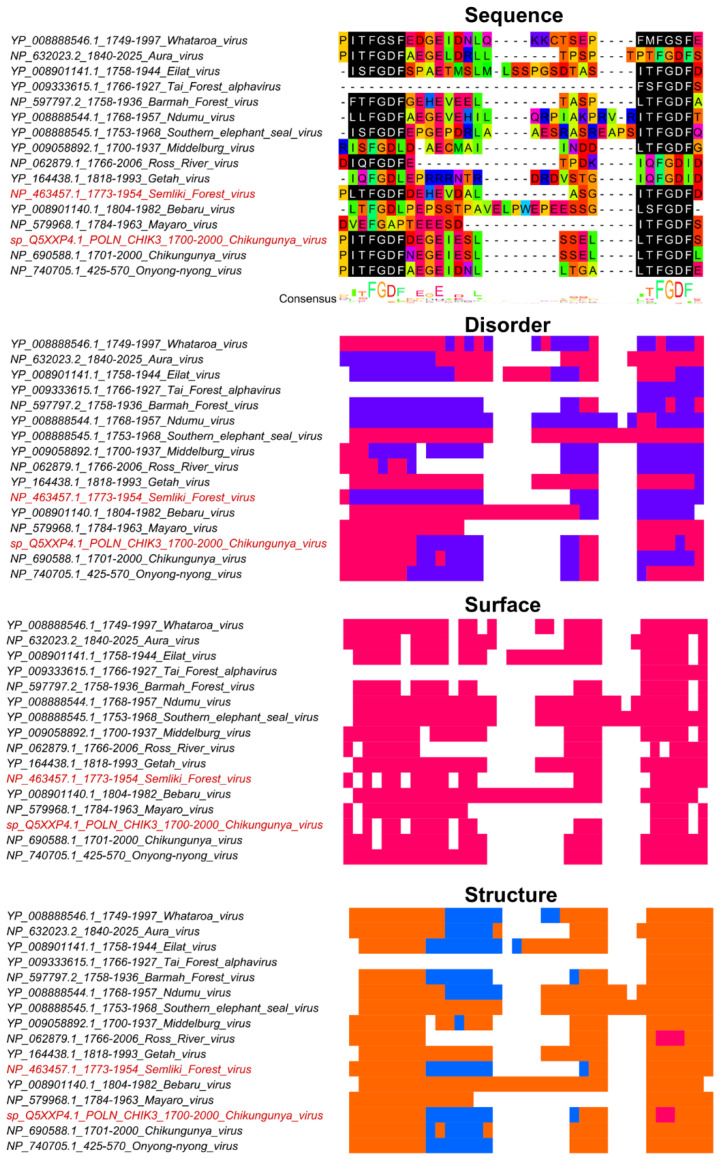
The G3BP binding motif has been verified in the nsp3 protein from Chikungunya virus and Semliki Forest virus from Alphaviruses. Sequences were identified with BLAST using residues 1700–2000 from nsp3 (accession: Q5XXP4) from Chikungunya virus as query. Sequence names shown in red represents true positive instances from the ELM database [27]. The multiple sequence alignment was built with MAFFT+L-INS-i [57] in Jalview [58]. The regular expression pattern [FYLIMV].FG[DES]F from motif LIG_G3BP_FGDF_1 in the ELM database [27] was identified using Find in Jalview, shown in black with white text. The region shown under Sequence shows the amino acids that corresponds to the true positive motifs from Chikungunya virus and Semliki Forest virus, the connecting amino acids, plus one additional site on each side. The MSA and heatmaps for Disorder, Surface, and Structure are colored as in Figure 3.

The multiple sequence alignment example shows a variation in the number of FGDF motifs among alphaviruses related to Chikungunya (Figure 4). Further, disorder and secondary structure is not conserved in this hypervariable region suggesting that functional divergence is likely for these FGDF motifs. For instance, in the Chikungunya virus the first motif is found to be in a completely disordered region and the second motif is lacking disorder in only one amino acid based on IUPRED predictions with a 0.4 cutoff. However, in the Semliki Forest virus, the two motifs were found to be in ordered protein regions.

In SARS-CoV-2, several studies have reported the interaction of nucleocapsid with the host G3BP proteins [73]. Upon interaction, attenuation of the host immune response occurs due to alteration of the process of stress granules inside the infected cells [74,75,76]. Kruse et al. proposed that the nucleocapsid-induced inhibition of stress granules is due to the presence of the ΦxFG pattern motif in nucleocapsid, where Φ means any hydrophobic residue, X means any amino acid and the last two amino acids in the motif are phenylalanine and glycine [77]. The motif in SARS-CoV-2 does not follow the last part of the pattern in the ELM database [DES]F, which suggests that the exact pattern is not essential for the functionality of the motif. 

### 4.3. SLiMs and Immune Cell Modulation 

**Figure 5:**
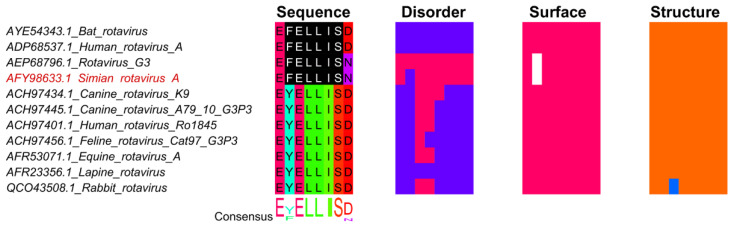
The pLxIS site in nsp1 from Simian rotavirus. Sequences were identified with BLAST using full-length nsp1 from Simian rotavirus (accession: AFY98633.1) as query. Sequence names shown in red represents true positive instances from the ELM database [27]. The multiple sequence alignment was built with MAFFT+L-INS-i [57] in Jalview [58]. The regular expression pattern [VILPF].{1,3}L.I(S) from motif LIG_IRF3_LxIS_1 in the ELM database was identified using Find in Jalview, shown in black with white text. The region shown under Sequence shows the amino acids that corresponds to the true positive motif from Simian rotavirus plus one additional site on each side. The MSA and heatmaps for Disorder, Surface, and Structure are colored as in Figure 3.

Viruses utilize diverse approaches to evade host immunity [78]. One strategy is the use of the pLxIS pattern by Rotaviruses [79]. In humans, the pLxIS motif is found in the stimulator of interferon genes (STING), mitochondrial antiviral signaling protein (MAVS), TIR domain-containing adaptor inducing IFN-β (TRIF), and in interferon regulatory factor 3 (IFN-3). Following the phosphorylation of the pLxIS motif in the adaptor proteins STING, MAVS, and TRIF, they interact with IFN-3 and stimulate the pLxIS motif’s phosphorylation in the transcription factor IFN-3. Next, detachment of the adaptor proteins occurs from the IFN-3 protein, followed by IFN-3 homodimerization and activation. Subsequently, the activated IFN-3 dimer transfers to the nucleus and activates the IFN-β gene’s transcription, triggering the release of INF-β from the infected cell and activating the innate immune response [79,80,81]. In Rotavirus, the pLxIS pattern is observed in the non-structural protein 1 (nsp1) and has the same affinity to IFN-3 as the adaptor proteins; however, when Rotavirus nsp1 pLxIS motif (Figure 5) binds to the IFN-3 protein, ubiquitination and degradation of IFN-3 are initiated. Hence, hindrance of IFN-β transcription occurs, and the virus can effectively escape host defense mechanisms and deactivate one of the innate immune responses [79,82].

### 4.4. SLiMs Modulating Host Cell Machinery

Although the previous steps are essential in the virus life cycle, viral proteins can also participate in other protein-protein interactions inside the host cell. Viruses can cause unfavorable cellular effects by mediating interactions with other cellular proteins. The following section shows how viruses use different viral-host PPIs to affect the pathogenicity and virulence of a diversity of viruses. 

#### 4.4.1. PDZ Binding Motif 

**Figure 6:**
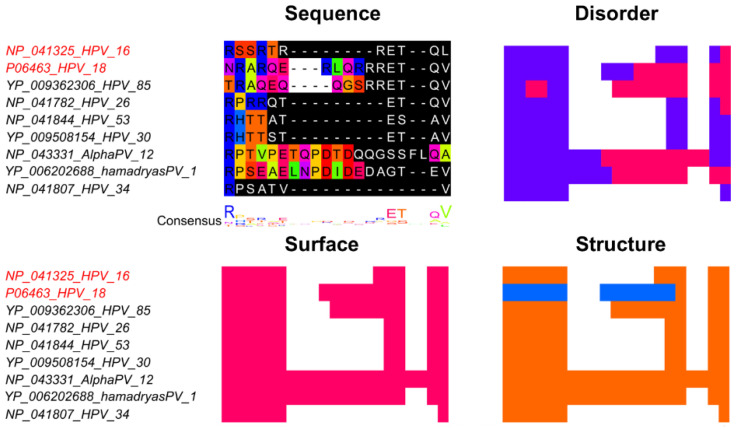
The PDZ domain binding motif in the E6 protein from HPV16 and HPV18. Sequences were identified with BLAST using protein E6 from HPV18 (accession: P06463.1) as query. Sequence names shown in red represents true positive instances from the ELM database [27]. The multiple sequence alignment (MSA) was built with MAFFT+L-INS-i [57] in Jalview [58]. The regular expression pattern …[ST].[ACVILF]$ from motif LIG_PDZ_Class_1 in the ELM database [27] was identified using Find in Jalview, shown in black with white text. The region shown under Sequence shows the amino acids that corresponds to the true positive motif from HPV16 and HPV18 plus one additional site on each side. The MSA and heatmaps for Disorder, Surface, and Structure are colored as in Figure 3.

PDZ domains are found in a vast number of proteins that recognize a specific C-terminal amino acid pattern [83]. According to the ELM database, the PDZ binding motif pattern is …[ST].[ACVILF]$ [27]. Proteins that include PDZ domains are involved in numerous cellular processes such as cell signaling pathways, subcellular transport, activating proteases, and recognizing misfolded proteins [83]. Hence, viruses that display a PDZ binding motif (PBM) will have the ability to bind to several PDZ domain containing proteins causing various effects depending on which PDZ domain they interact with [84]. Oncogenic human adenovirus 9 E4 protein and human papillomavirus 18 E6 protein include a PDZ binding motif in their C-terminal regions. Both proteins bind to PDZ domain containing proteins MUPP-1, Dlg, and MAGI-1 [85]. MUPP-1, a multi PDZ domain protein that comprises 13 PDZ domains, is an essential protein for maintaining cell polarity at the tight junction [86]. Dlg, a Drosophila discs large protein and a protein with 3 PDZ domains, is one of the scribble complex proteins, which are involved in maintaining the cellular polarity and adhesion at the cellular junction [87]. MAGI-1 is a membrane associated guanylate cyclase that is located in cellular junction and is important for regulating the proliferation and cellular adhesion between cells [88]. Dlg and MAGI-1 function in tumor suppression [85,87,88]. The binding of human adenovirus 9 to these human proteins inhibits their function through sequestration. Adversely, the E6 protein of some human papillomavirus (HPV) strains that includes the PBM in its C-terminal region will induce these proteins breakdown [85]. Infections with human papillomavirus strains containing PBM in the E6 protein pose a higher risk of causing HPV-associated metastatic cancer. Through the PBM, the E6 protein can perform an interaction with the cellular polarity proteins, leading to loss of cellular polarity and promotion of the proliferation and invasion of cancerous cells [89,90]. The multiple sequence alignment shows that this SLiM is in a highly varying region (Figure 6). The sequence diversity in this region makes it difficult to make a good multiple sequence alignment. Further, intrinsic disorder prediction suggests that this SLiM is not consistently in a disordered region, but the surface accessibility is consistent. Interestingly, the first half of the motif in HPV18 is structured (helix) but the remaining part of the motif is found in a coil state. Such variations may be due to inaccurate predictions but could also be a symptom of functional divergence between the PDZ binding motifs.

In SARS-CoV, the envelope protein was found to include PBM, which has the ability to interact with the PDZ domain in the syntenin protein. The interaction of SARS-CoV envelope protein with syntenin was correlated with the P38 MAPK activation, inducing the production of inflammatory cytokines. Mutant PBM motif was correlated with decreased inflammatory response in SARS-CoV infected mice [91]. However, other studies showed that the PBM found in both SARS-CoV and SARS-CoV-2 envelope proteins is capable of interacting with PALS1 protein which is important for maintaining cellular polarity at the cell junction [92,93,94]. The PBM motif in the envelope protein from SARS-CoV and SARS-CoV-2 has the sequence DLLV [94], which resembles the LIG_PDZ_Class_2 pattern in the ELM database (…[VLIFY].[ACVILF]$) [27], and was found to be in a structurally flexible region that resembles the C-terminal unstructured region in Crumbs protein (Crb-CT). Crb and PATJ protein (PALS1-associated tight junction) binds to PALS1 to form the Crumbs Cell Polarity Complex Component, which is responsible for maintaining cell polarity at the cellular junction [94]. Both the C-terminal BPM motif and the Crb-CT region of the envelope protein were found to bind to PALS1 in a similar fashion [94]. However, the interaction between the envelope protein and PALS1 is thought to cause alteration in the subcellular location of PALS1. The re-localization of the PALS1 protein to where virus is assembled impedes the cellular junction protein complex formation in the infected epithelial cells. Thus, the infected cell will lose its polarity which can facilitate the viral release from the cells [92,93,94]. 

#### 4.4.2. The 14-3-3 Domain-Binding Motif 

Another common viral-host interaction is mediated through Serine and Threonine (ST) rich motifs in the 14-3-3 protein family. 14-3-3 proteins are involved in a myriad of signaling pathways and interact with numerous cellular proteins [95,96,97]. The interaction of the 14-3-3 protein depends on the phosphorylation state of the binding motif. Thus, kinases and phosphatases can affect the motif’s binding to the 14-3-3 protein [98]. SLiM mediated binding to 14-3-3 proteins can (1) induce structural changes, (2) block the active site, (3) facilitate the interaction between the motif-containing protein and other proteins, or (4) alter the cellular location of the binding partner [97,98]. 

In Hepatitis C virus (HCV), the HCV core protein interaction to 14-3-3 protein activates the kinase Raf-1, which induces cellular proliferation and abnormal growth [99]. The HCV genotype 1b core protein has been reported to interact with Raf-1 kinase using the sequence motif RKTpSER, and the phosphorylation of the serine residue was found to be essential for the motif activity [99]. This sequence motif partially overlaps with the R[^DE]{0,2}[^DEPG]([ST])(([FWYLMV].)|([^PRIKGN]P)|([^PRIKGN].{2,4}[VILMFWYP])) pattern of the canonical 14-3-3 binding motif (LIG_14-3-3_CanoR_1) in the ELM database [27]. 

In SARS-CoV, binding of the 14-3-3 domain-containing proteins to the phosphorylated nucleocapsid is involved in translocation of the nucleocapsid protein between the cytoplasm and nucleus, altering the functionality of the 14-3-3 interacting protein [100]. In the closely related SARS-CoV-2, nucleocapsid has not yet been detected in the nucleus, but it has been found to interact with various 14-3-3 protein isoforms in the cytoplasm [101]. Several sequence patterns identified in both SARS-CoV and SARS-CoV-2 are found in a disordered S/T-rich protein region of the nucleocapsid protein with multiple known phosphorylation sites [101] and resemble, either partially or completely, the canonical 14-3-3 pattern found in the ELM database [27]. The phosphorylation and disorder property of the presented motifs suggest a similarity to other 14-3-3 binding motifs where phosphorylation and disorder are essential for interacting with the 14-3-3 domain-containing proteins [102]. Although no viral 14-3-3 binding motif examples are included yet in the ELM database, these examples highlight that viruses may have numerous molecular effects on cells through interactions with 14-3-3, mediated by SLiMs.

### 4.5. SLiMs Responsible for Viral Exit from the Cell

**Figure 7:**
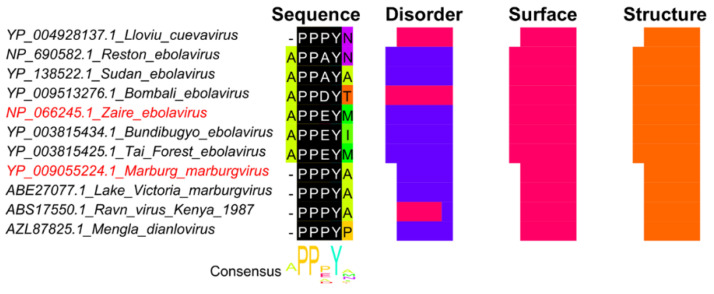
The PPxY motif in the matrix protein VP40 from Ebola virus. Sequences were identified with BLAST using full-length VP40 from Ebola virus (accession: Q05128) as query against the refseq_protein and nr databases. Sequence names shown in red represents true positive instances from the ELM database [27]. The multiple sequence alignment was built with MAFFT+L-INS-i [57] in Jalview [58]. The regular expression pattern PP.Y from motif LIG_WW_1 in the ELM database [27] was identified using Find in Jalview, shown in black with white text. The region shown under Sequence corresponds to the true positive motif from Zaire Ebola virus and Marburg marburg virus plus one additional site on each side. It should be noted that query protein Q05128 Uniprot ID is identical to protein NP_066245.1 used in the multiple sequence alignment.

Viruses have several strategies to egress their host cells, which can be achieved through cell lysis, budding from the cell membrane, or exocytosis using the secretory pathway. SLiMs can enhance viral egress through budding. One example is the interaction of the viral proteins with the endosomal sorting complexes required for transport (ESCRT) pathway inside the cell. The importance of viral late domains (L domains) has been widely implicated in the viral budding process, and short sequence motifs, P[TS]AP, PPxY, and LYPxL, have been involved in the interaction with the ESCRT pathway machinery [103,104,105]. Such motifs were found to be highly conserved across diverse types of viruses, including Poxviruses [106], Hepatitis C viruses [107], Rhabdoviruses [108], Retroviruses [109], Arenaviruses [110], and Filoviruses [111,112]. Ebola VP40 (Figure 7) and HIV-1 contain PPxY motifs that are recognized by a highly conserved enzyme in humans (E3 ubiquitin ligase) [113,114]. E3 ubiquitin ligase enzyme is involved in regulating a plethora of biological processes by stimulating the ubiquitination and subsequent degradation of their target protein [115]. Interactions with the WW domain of ubiquitin ligase enzymes, recruitment of Tsg101, and the ubiquitination by specific ubiquitin ligase enzymes have been shown to facilitate the ESCRT pathway-mediated viral budding [113,114]. The role of ESCRT pathway and viral late domains in viral exit have been extensively reviewed [103,116], including the importance of the ESCRT pathway in different phases of the viral life cycle [117].

## 5. Conclusions and Future Perspective

The small genome size of viruses and their inability to replicate outside a host go hand in hand with their need to hijack host cell machinery [2]. SLiMs with varying evolutionary rates in different viral families can mutate to accommodate various selective pressures stemming from their environment. The fitness of viruses depends on their capacity to alter host cell machinery and escape detection by the immune system. This capacity is governed, in part, by the potential to mimic and compete with functionally important protein interactions. In this review, we highlighted the importance of viral mimicry mediated by SLiMs at select steps of the virus life cycle. We also showed how specific SLiMs might affect virulence and pathogenicity. These SLiM actions are mediated by viral-host protein-protein interactions. 

Previous studies on eukaryotic SLiMs showed that physicochemical properties, such as secondary structure and disorder, should be considered when studying SLiMs as the majority of the functionally verified SLiMs were found to be disordered and enriched with polar residues [34]. Based on disorder predictions, the true positive experimentally verified viral SLiMs deposited in the ELM database are not necessarily intrinsically disordered, but they are surface exposed and mainly in a conformationally flexible coil rather than in alpha helices or beta strands. Our findings for the viral SLiMs give rise to questions regarding disorder content and other structural characteristics of the corresponding eukaryotic linear motifs in the hosts of viruses, and for eukaryotic linear motifs, in general. The ELM database has grown rapidly over the last 10 years and re-analysis of disorder content is warranted. Among the viral SLiMs, the most abundant categories are the ligand binding sites and post-translationally modified sites. Ligand binding sites are the most common class among the fully disordered sites, while the post-translational modification sites are the most common among the fully ordered sites. Given that disorder content appears to vary between different functional classes of motifs, an analysis into disorder content variation across these classes may illuminate function-specific traits of importance in differentiating false and true positive SLiMs.

Proteomes from eukaryotes tend to have more disorder content overall than proteomes from bacteria and viruses [118,119]. It is possible that the disorder content required for SLiMs to be functional not only depends on the identity of the SLiM, but also on other contexts such as genome complexity and overall disorder content of the proteome. Eukaryotic genomes evolve under multifaceted constraint that differ from the constraint acting on viruses [120]. For eukaryotes, disordered regions are often able to participate in multiple distinct PPIs [121]. Disorder is advantageous at binding interfaces that rely on conformational transitions where SLiMs controlled by post-translational modifications may act as molecular on/off switches [122]. However, disorder may become less advantageous when an ordered viral SLiM mimics a functional conformation of a host SLiM so that it is always switched on or off.

We showed an example of the G3BP binding motif in Chikungunya virus and Semliki Forest virus. Based on IUPRED, the former is found in a disordered region, while the latter is in an ordered region (Figure 4). Since intrinsic disorder is not conserved, changes in disorder can potentially change the functional potential of a SLiM; however, intrinsic disorder may not be as important for viral SLiMs as often stated. The majority of the experimentally confirmed viral SLiMs were almost entirely found in a surface accessible coil region, unlike disorder where at least 1 in 4 motifs was devoid of disorder. HIV-1 envelope furin cleavage site motif and E6 HPV 18 PBM were predicted to be a mix of both coil and helix, which poses a question about the differences between flexible and disordered protein regions, and whether flexibility and disorder should both be considered when searching for functional SLiMs. 

**Figure 8:**
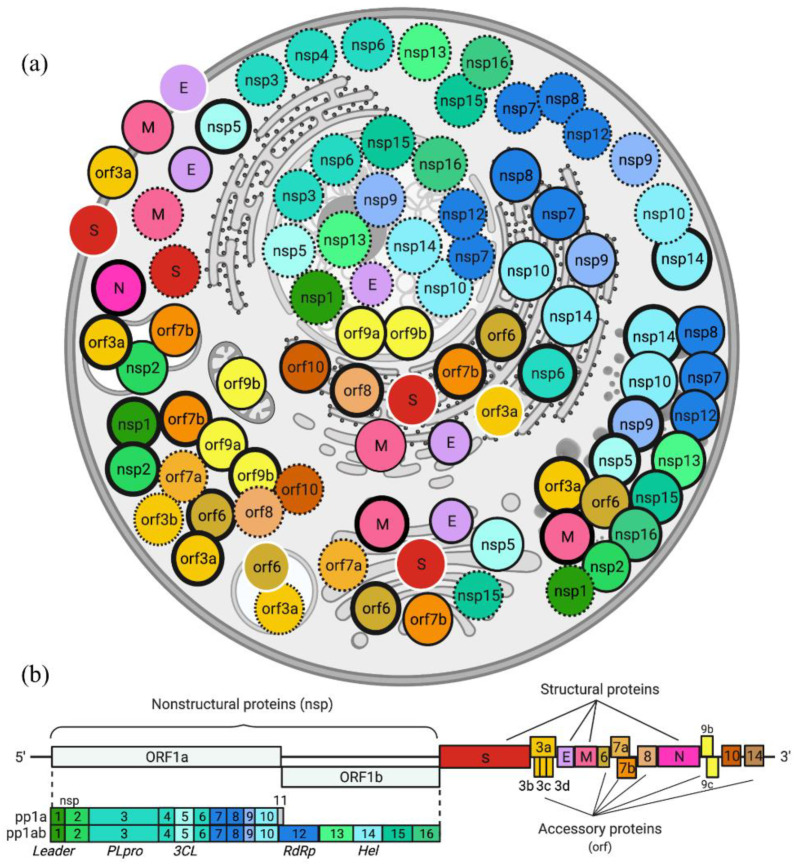
Cellular context. Subcellular localization of SARS-CoV-2 proteins (circles) in human cells based on experimental data (thick border: multiple sources; dotted border: [127]; thin black border: [128]; white border: [129,130,131]). (**a**). Each protein is colored as in the SARS-CoV-2 proteome (**b**). Proteins that form complexes are colored similarly; nsp 3/4/6, nsp 7/8/12, nsp 10/14. SARS-CoV-2 proteins localize to the following organelles: lysosome (nsp2, orf3a, and orf7b), endosome (orf3a and orf6), plasma membrane (envelope (E), membrane (M), spike (S), and orf3a), Golgi apparatus (E, M, S, nsp5, nsp15, orf6, orf7a, and orf7b), endoplasmic reticulum (E, M, S, nsp6-10, nsp14, orf6, orf7b, orf8, and orf10), nucleolus (E, nsp1, nsp3, nsp5-7, nsp9-10, nsp12-16 and orf9a-9b), punctate cytoplasm (M, nsp1, nsp2, nsp5, nsp7-10, nsp12-16, orf3a, and orf6), and diffuse cytoplasm (E, M, nucleocapsid (N), S, nsp1-16, nsp10, nsp12-16, orf3a-3b, orf6, orf7a-7b, orf8, orf9a-9b, and orf10). Created with BioRender.com (accessed on 30 October 2021).

Experimental verification of viral SLiMs can be challenging. The large SARS-CoV-2 dataset that has accumulated since this virus emerged in late 2019 has a wealth of information. Currently, some SLiMs for SARS-CoV-2 have been verified [123,124]. We expect that more are to come and that they will contribute to how we analyze viral SLiMs. For example, the subcellular location of most SARS-CoV-2 proteins have been determined (Figure 8). The Cell Atlas [125] and the Human Protein Atlas [126] provide subcellular locations and more for human proteins. Combining information about shared cellular locations will further illuminate potential viral network interference in the host cell. Computational methods provide time- and cost-effective, low-risk ways to predict the presence and function of these crucial motifs, which may be experimentally verified in vitro. 

While the limitations of both computational and experimental approaches of linear motifs must be closely considered to decrease the probability of misleading false positive results, predictions of SLiMs have proven helpful in elucidating how SARS-CoV-2 interacts with its human host (e.g., [44,61,128,132]). Altogether, this review shows the promise for how molecular mimicry discovery in different viral families can improve our understanding of the virus-host interface. 

## Data Availability

Data included in supplementary materials.

## Supplementary Materials

The following are available online at https://www.mdpi.com/article/10.3390/v13122369/s1, Table S1: viral_slim_predictions.csv.
